# Association of the gut microbiome to colorectal anastomotic leakage: systematic review

**DOI:** 10.1093/bjsopen/zrag005

**Published:** 2026-04-08

**Authors:** Peter H Cashin, Sara Artursson, Filip Sköldberg, Åsa Melhus

**Affiliations:** Department of Surgical Sciences, Uppsala University, Uppsala, Sweden; Uppsala University Hospital (Akademiska sjukhuset), Uppsala, Sweden; Department of Surgical Sciences, Uppsala University, Uppsala, Sweden; Uppsala University Hospital (Akademiska sjukhuset), Uppsala, Sweden; Department of Surgical Sciences, Uppsala University, Uppsala, Sweden; Uppsala University Hospital (Akademiska sjukhuset), Uppsala, Sweden; Uppsala University Hospital (Akademiska sjukhuset), Uppsala, Sweden; Department of Medical Sciences, Section of Clinical Microbiology, Uppsala University, Uppsala, Sweden

**Keywords:** surgical site infection, dehiscence, bacteria, cancer, alpha, beta

## Abstract

**Background:**

Research into the gut microbiome and its possible association with anastomotic leakage after colorectal surgery has increased recently with the growing availability of sequencing techniques. There is a lack of systematic reviews addressing specifically microbiomic differences between patients with anastomotic leakage and patients with a successful anastomotic healing. The objective was to systematically review the current research on the microbiome and its effect on the risk of anastomotic leakage in colorectal cancer.

**Methods:**

Pubmed/Medline, Cochrane, and Google scholar were searched on 14th February 2025, to identify relevant publications with the following inclusion criteria: colorectal surgery, microbiome sequencing data, anastomotic leakage as endpoint, and comparative groups. Exclusion criteria were studies conducted exclusively on animals, non-peer-reviewed studies, review articles, and unavailable full text. Alpha/beta diversity and microbiomic functional analyses were the focus of the results.

**Results:**

From 112 studies, 11 studies including 551 patients were included: 143 patients with anastomotic leakage and 408 as controls. Alpha diversity differences were found in 7 of 11 studies—1 of 4 with preoperative sampling versus 6 of 7 studies with intra/postoperative sampling (*P* = 0.044). Beta diversity differences were found in 5 of 11 studies. Three studies reported on functional analyses, with one study demonstrating an association between methanogenesis and anastomotic leakage. Bacterial abundance was inconsistent across the studies. Three studies involving rodent models indicated a causal effect of the clinical microbiome.

**Conclusion:**

Evidence implicates the gut microbiome as a factor associated with anastomotic leakage in colorectal cancer surgery, with three studies suggesting a causal relationship. There is a shortage of studies evaluating cross-species functional profiling. Optimal sampling should be performed during surgery.

## Teaser

Differences in microbial diversity and bacterial abundance in the gut microbiome are associated with anastomotic leakage in colorectal cancer surgery. Optimal timing of faecal sampling is unknown. Functional profiling of bacteria and the association with anastomotic leakage is unknown. The association of microbial diversity and bacterial abundance to anastomotic leakage is confirmed in this systematic review, albeit not in consistent ways. This review identifies the faecal sampling timepoint as significant for determining differences in the microbiome between patients with anastomotic leakage and those without. There is a significant shortage of functional profiling studies and study heterogeneity is a major obstacle in finding common ground for future studies. Future studies should focus on intraoperative faecal sampling for optimal diversity comparisons and more cross-species functional profiling in a more homogenous clinical setting with larger sample sizes.

## Introduction

Anastomotic leakage (AL) is a severe complication following colorectal surgery, leading to increased morbidity and mortality. Despite advancements in surgical techniques, AL incidence remains between 4 and 15%^1^. This complication arises when the integrity of a surgically created anastomosis is compromised, allowing the contents of the gastrointestinal tract to leak into the abdominal cavity. This not only delays recovery but also increases the risk of sepsis and death^2,3^.

Mortality from postoperative bowel infections is expected to rise, as resistance to carbapenems has increased more than to any other major antibiotic class in recent years^4,5^. Intestinal colonization with bacteria resistant to these agents is an established risk factor for severe, potentially life-threatening infections, as demonstrated in a recent case–control study^5^ comparing carbapenem-resistant with carbapenem-sensitive infections.

Apart from being a reservoir of antibiotic resistance genes, emerging evidence implicates the gut microbiome as a critical factor influencing anastomotic healing. The human gastrointestinal tract hosts trillions of microorganisms playing essential roles in immunity, metabolism, and maintaining intestinal barrier integrity. Dysbiosis, or an imbalance in the gut microbiome, has been identified as a potential contributor to poor anastomotic healing^6–9^. Mechanistic studies suggest that certain microbial communities can either promote or hinder tissue repair and immune modulation. Members of the Lachnospiraceae family and the genus *Fusobacterium* have been associated with adverse surgical outcomes, including AL, whereas bacteria such as streptococci may exert protective effects^6–9^.

There have been several advancements in this research field in recent years, and on microbiome functional analyses in particular^10–15^. The functions of a microbiome are often more important than the species included, as different bacteria can have similar or even identical functional roles. This means that, despite being taxonomically distinct, they can perform the same metabolic functions or contribute similarly to a particular environment. The advancement in this area prompted this systematic review, in which current evidence on the association between gut microbiome composition and AL is synthesized. Unlike traditional culture-based methods, sequencing provides a comprehensive and unbiased view of microbial communities, including unculturable organisms that may influence anastomotic healing.

The review focuses on microbial diversity, specific taxa, and functional analyses, and explores potential future research strategies for mitigating AL risk.

## Methods

A systematic review was undertaken according to the PRISMA 2020 guidelines using the eligibility criteria below.

### Eligibility criteria

Studies were included if they:

- Reported microbiome sequencing data related to colorectal surgery.
- Defined AL as a primary endpoint.
- Provided full-text access in peer-reviewed journals.
- Had comparison groups of AL and controls.

Studies were excluded if they:

- Were reviews, book chapters, or commentaries.
- Focused exclusively on animal models without human data or human faecal transplants.
- Did not perform microbiome sequencing.

Sequencing-based microbiome analysis was an explicit inclusion criterion because it enables standardized assessment of alpha and beta diversity, microbial abundance, and functional pathways—key outcomes relevant to this review. Studies relying solely on culture-based or non-sequencing techniques were excluded to ensure methodological comparability and to avoid introducing bias from incomplete microbial detection. Furthermore, the type of sequencing technology used (for example 16S ribosomal ribonucleic acid (rRNA) *versus* shotgun metagenomics) was extracted to contextualize interstudy variability. This approach allowed a consistent evaluation of microbiome profiles across the included studies.

### Search strategy

**Fig. 1:**
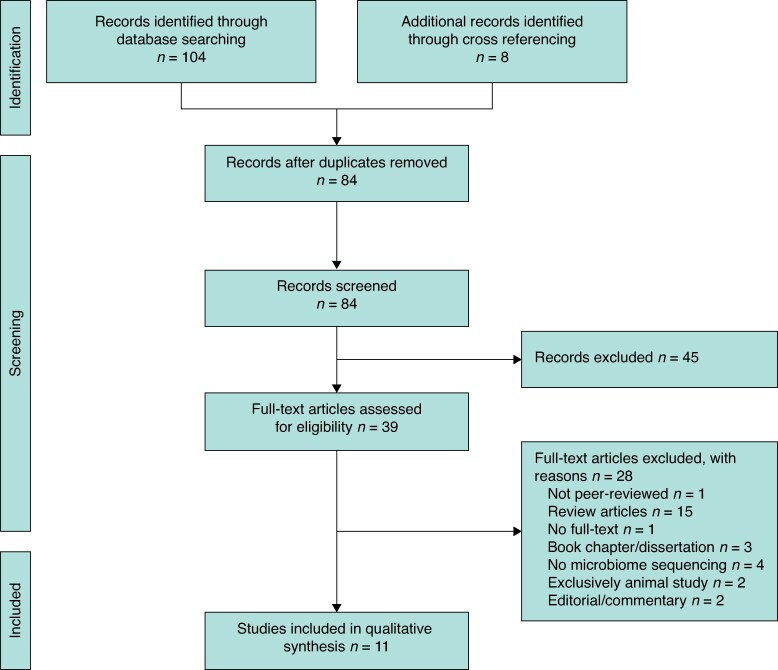
PRISMA diagram of the study selection process

The search was performed on 14 February 2025. For the Google Scholar search, the following keywords were used: (‘colorectal surgery’ OR ‘colon surgery’ OR ‘rectal surgery’ OR ‘anastomosis’) AND (‘gut microbiota’ OR ‘gut flora’ OR ‘intestinal microbiome’ OR ‘microbial biomarkers’) AND (‘anastomotic leakage’ OR ‘postoperative complications’ OR ‘surgical outcomes’) AND (‘predictive biomarkers’ OR ‘prognostic biomarkers’). A total of 55 studies were identified. The same keywords and search criteria were applied in Pubmed, but the last keywords (AND (‘predictive biomarkers’ OR ‘prognostic biomarkers’)) were omitted to render search results. The Pubmed search resulted in 36 titles. A search in the Cochrane database using the same keywords as the Pubmed search found 13 studies. After the initial search, cross-referencing was performed. This yielded eight more articles. Of the 112 articles found, 73 articles were excluded in a first screening and another 28 after full text screening (see *Fig. 1* for details). The final 11 studies and their references were reviewed, and it was assessed as probable that all relevant published articles had been captured in the screening process.

### Data extraction and synthesis

Two independent reviewers (P.C. and S.A.) extracted the following data from the included studies: number of patients, number of leaks in patients *versus* in control patients, matching processes, method of sequencing, faecal sample type, type of colorectal surgery, and type of anastomosis. Furthermore, outcome measures were collected for microbial diversity defined as alpha and beta diversity, for specific taxa in relation to differences in abundance between AL patients and controls, profiling of specific bacterial functional pathways, and, lastly, causal testing in rodent models. Discrepancies were resolved by consensus. Study bias was evaluated using the Newcastle–Ottawa scale for non-randomized study comparisons. Due to the nature of the outcome measures, narrative specifications were used, and meta-analyses were not applicable. The review was registered in the PROSPERO database (CRD420251018751).

This is a systematic literature review that does not require any ethical approval. Furthermore, the aim of this review is ethically sound.

## Results

### Study population and bias evaluation

The 11 included studies encompassed 551 patients, of whom 143 had AL and 408 served as controls^6,10,11,13–20^. As a rule, the studies were limited in patient size (median 20 (range 16–162)). Male patients predominated. The type of sample chosen for exploring the microbiome displayed a wide range of variation (see *Table 1* for a summary of the patient-related data).

**Table 1:** Summary of patient-related data from the included studies

Study	No. of patients	No. of leaks	No. of controls	Matched groups	Type of sample	Type of surgery	Type of anastomosis
van Praagh *et al.*^19^	16	8	8	1:1 matching (NR)	Doughnut ring after circular-stapled anastomosis	Resection with colorectal anastomosis	Stapled—circular
van Praagh *et al.*^16^	123	29	94	Unclear matching (NR)	Doughnut ring after circular-stapled anastomosis	Resection with colorectal anastomosis	Stapled—circular
Shogan *et al.*^18^	101	15	86	None performed—all included	Rectal swab intraoperative and rectal swab or stoma swab POD2	Hemicolectomy (45), proctectomy (36), colectomy (5), APR (13), other (2)	NS
Palmisano *et al.*^6^	21	5	16	None performed—all included	Faecal—preoperative	Right hemicolectomy (12), left hemicolectomy (1), anterior resection (8)	NS
Shi *et al.*^20^	20	10	10	1:1 matched controls (NR)	Surgical biopsy	NS	NS
Jin *et al.*^17^	34	17	17	1:1 Propensity score matching (NR)	Faecal—first postoperative defecation	NS	NS
Hoedt *et al*.^15^	162	25	137	None performed—all included	Intraoperative swab	Hemicolectomy (74), colectomy (3), resection with colorectal anastomosis (77)	NS
Hernández-González *et al.*^14^	20	10	10	1:1 matched controls (101)	Surgical biopsy/faecal	Right hemicolectomy (52), left hemicolectomy (12), sigmoidectomy (47)	Staples + handsewn (52%), staples (28%), handsewn (20%)
Hajjar *et al.*^11^	18	9	9	1:1 matched controls (68)	Preoperative stool	NS	NS
Wang *et al*.^13^	20	10	10	1:1 propensity score matching (88)	Faecal—preoperative	Hemicolectomy (9), rectal resection (11)	NS
Lehr *et al.*^10^	16	5	11	None performed—all included	Endoscopically 4 days before surgery	Anterior resection	NS

Values are *n* unless otherwise stated. NR, not reported; POD, postoperative day; APR, abdominoperineal resection; NS, not stated.

In the bias evaluation, the studies received scores of five to seven stars out of a maximum of nine. Four studies (36%) scored seven stars (*Table 2*).

**Table 2:** Study bias evaluation according to the Newcastle–Ottawa scale

Study	Selection (0–4 stars)	Comparability (0–2 stars)	Exposure (0–3 stars)	Total
van Praagh *et al.*^19^	3	1	2	6
van Praagh *et al.*^16^	3	1	2	6
Shogan *et al.*^18^	3	1	1	5
Palmisano *et al.*^6^	3	1	2	6
Shi *et al.*^20^	3	1	2	6
Jin *et al*.^17^	3	2	2	7
Hoedt *et al.*^15^	3	1	2	6
Hernández-González *et al*.^14^	3	2	2	7
Hajjar *et al.*^11^	3	2	2	7
Wang *et al*.^13^	3	2	2	7
Lehr *et al.*^10^	3	1	2	6

Values are *n*.

### Microbiological findings

All studies used 16S rRNA for sequencing. The V3–V4 region was most favoured as the target. One study included, in addition, metagenomics shotgun sequencing, whereas one study did not provide any information on the region of sequencing (*Tables 3*, *4*).

**Table 3:** Summary of diversity and functional findings from the included studies

Study	Region targeted with 16S rRNA + other sequencing method	Functional analysis	Differences in alpha diversity	Differences in beta diversity
van Praagh *et al*.^19^	V3–V4	NA	Yes (Simpson)	No
van Praagh *et al.*^16^	V3–V4	NA	Yes (Simpson)	No
Shogan *et al.*^18^	V3–V4	NA	No	No
Palmisano *et al.*^6^	Data missing	NA	Yes (Simpson, and Chao)	No
Shi *et al.*^20^	V3–V4	Matrix metalloproteinase 9 activation	Yes (Simpson, Shannon)	Yes
Jin *et al.*^17^	Data missing	EMT, collagen synthesis, *TGF-β/Smad* increase anastomotic healing	Yes (Sobs)	Yes
Hoedt *et al.*^15^	V3–V4 + metagenomic shotgun sequencing	Methanogenesis increased in AL	Yes (Simpson, Shannon)	Yes
Hernández-González *et al.*^14^	V3–V4	NA	Yes (Shannon)	No
Hajjar *et al.*^11^	V5–V6	*P. goldsteinii* improved anastomotic healing by suppressing the proinflammatory *MyD88/NF-κB* signalling pathway	No	Yes
Wang *et al.*^13^	V3–V4	NA	No (Simpson, Shannon, Sobs, Chao, Ace)	Yes
Lehr *et al*.^10^	V1–V2	NA	No for AL, yes for longitudinal postoperative differences	No

rRNA, ribosomal ribonucleic acid; NA, not available; EMT, epithelial to mesenchymal transition; AL, anastomotic leak.

**Table 4:** Summary of abundance and causality findings from the included studies

Study	Differences found in abundances	Increased abundance in AL	Decreased abundance in AL group/higher in control group	Causality tested in animal model
van Praagh *et al*.^19^	Yes	Lachnospiraceae, Bacteroidaceae	None	NA
van Praagh *et al.*^16^	Yes	*Bacteroides,* Lachnospiraceae*, Blautia*	*Prevotella, Streptococcus, Eubacterium*	NA
Shogan *et al.*^18^	No	None	None	NA
Palmisano *et al.*^6^	Yes	*Acinetobacter lwoffii, Acinetobacter johnsonii, Hafnia alvei*	*Barnesiella intestinihominis, Faecalibacterium prausnitzii*	NA
Shi *et al.*^20^	Yes	Lachnospiraceae, Bacteroidaceae, Fusobacteriaceae, *Fusobacterium nucleatum*	None	Causality successfully tested in rat model for Fusobacteriaceae
Jin *et al.*^17^	Yes	*Romboutsia, Blautia, Bacteroides, Clostridium sensi stricto, Ruminococcus gnavus, Eggerthella*	*Lactobacillus, Comamonas*	Causality demonstrated in rat model for AL, faecal transplants
Hoedt *et al.*^15^	Yes	*Aeromonas, Butyrivibrio, Colidextribacter, Lachnospira, Sutterella*	*Bacteroides, Butyrivibrio, Citrobacter, Gemella, Klebsiella, Lactobacillus, Streptococcus*	Mouse model developed but causality not tested
Hernández-González *et al.*^14^	Yes	None	*Actinobacteria, Pseudomonas*	NA
Hajjar *et al.*^11^	Yes	15 sequence variants (specific interest—*Alistipes onderdonkii*)	13 sequence variants (specific interest—*Parabacteroides goldsteinii*)	Causality demonstrated in mouse model, oral supplementation with bacteria
Wang *et al.*^13^	Yes	None	*Proteobacteria, Streptococcus, Citrobacter, Klebsiella*	NA
Lehr *et al*.^10^	Yes	*Prevotella, Faecalibacterium*	*Phocaeicola, Ruminococcus*	NA

AL, anastomotic leak; NA, not available.

#### Microbial diversity

- Alpha diversity: seven studies^6,14–17,19,20^ reported lower alpha diversity in patients with AL, indicating reduced microbial richness and evenness. Among these studies, one of four (25%) involved preoperative sampling, whereas six of seven (86%) included intra- or postoperative sampling (*P* = 0.044).
- Beta diversity: five studies^11,13,15,17,20^ found significant compositional differences between the AL and control groups, highlighting distinct microbial community structures.

#### Specific taxa

- Increase in AL: Lachnospiraceae were reported as elevated in patients with AL but not in the controls in four studies^12,15,19,20^.
- Decrease in AL: streptococci and *Citrobacter* were identified as protective taxa with reduced AL in three studies^12,16,19^. Lactobacilli were identified in the control group as protective in two studies^15,17^.
- Unclear: other taxa were associated with AL (*Table 4*), but their abundance varied across studies, either increasing or decreasing in relation to AL, making it difficult to determine their relevance.

#### Functional profiling

There was a considerable lack of functional profiling in the included studies. However, in the only study^15^ utilizing metagenomic shotgun sequencing it was reported that pathways leading to methanogenesis were elevated in patients with AL.

Although full functional profiling was not performed, three other studies explored specific functional pathways in rodent models. One^17^ of them demonstrated that epithelial–mesenchymal transition, collagen synthesis, and *TGF-β*/*Smad* were associated with improved anastomotic healing. Another^11^ investigated only a specific bacterium, *Parabacteroides goldsteinii*, and concluded that it enhanced anastomotic healing by suppressing the proinflammatory *MyD88/NF-κB* signalling pathway, a major player in the innate immune response. The third study^20^ had a similar approach and examined the causative role of *Fusobacterium nucleatum* in AL. Upregulation of the expression of matrix metalloproteinase 9, leading to a collagen depletion, was reported as a probable mechanism of action.

### Experimental causative evidence

The three studies^11,17,20^ that examined specific functional pathways also demonstrated that bacteria associated with AL may exert causative effects in rodent models. One study^11^ explored how exposure to the two Gram-negative anaerobic rods. *Alistipes onderdonkii* and *P. goldsteinii*, modulated the anastomotic healing. Oral supplementation with *A. onderdonkii* led to higher leak rates, whereas gavage with *P. goldsteinii* resulted in improved anastomotic healing in mice. A second study^17^ used faecal transplants from patients with or without AL. The gut microbiota from patients without leakage improved the anastomotic healing in a rat anastomotic model. Finally, in a third study^20^, *F. nucleatum* was identified as a potential biomarker for AL and used to demonstrate its role in inducing clinical leaks in a rat model.

## Discussion

This systematic review highlights differences in gut microbiome composition between patients with AL and those without complications. Whereas some studies reported consistent findings regarding certain bacteria, a major issue of inconsistency across studies remained evident. Alpha and beta diversity metrics revealed reduced microbial richness and distinct community structures in patients with AL. Additionally, experimental evidence supports a causal relationship, as faecal transplants from patients without AL improved anastomotic healing in animal models. These findings offer opportunities for future research that may identify key microbial biomarkers and therapeutic targets to reduce AL risk. However, achieving this requires harmonizing methodologies and compiling more comprehensive data on both microbiome and functional profiles from patients living in different geographical areas and with different dietary and environmental exposures.

Alpha diversity, investigating the diversity within a sample, differed between AL and control groups in 7 of 11 studies, showing reduced microbial richness in the AL samples. Interestingly, stratifying according to when the sample was taken, before surgery or during/after surgery, revealed that alpha diversity differences were more often reported as statistically significant in the intra/postoperative sampling setting (25% *versus* 86%). Possible explanations for this could be the effects of mechanical bowel preparations and/or preoperative antibiotic treatment, especially in malnourished patients with a more easily disrupted gut microbiota. Patients who retained greater microbial diversity after preoperative antibiotics had a reduced risk of AL, whereas those who experienced a decrease in microbial diversity faced a higher risk of AL. Alpha diversity may not be an important factor in the preoperative setting, whereas it appears to gain importance in the operative or postoperative setting. Unfortunately, it is not logistically feasible to test alpha diversity during surgery as a biomarker for AL, but this finding supports the suggestion that probiotic treatment could be a way to prevent reduced microbial richness due to preoperative antibiotics, which for many reasons still need to be administered and have also been shown to reduce the risk of AL^21^.

This review is the first one to find an interstudy difference in alpha diversity dependent upon when sampling was performed, as detailed above. Moreover, the findings in this review are not concordant with those in the review by Jørgensen *et al.*^7^, which associated collagenase-producing bacteria such as *Enterococcus faecalis* with AL. The key difference between the two reviews lies in the preferred methodology of the included studies: Jørgensen *et al.* incorporated data from cultured bacteria, whereas this review used sequencing data. The review by Lianos *et al.*^8^ is, however, more aligned with this one and emphasizes reduced microbial diversity in AL patients. It shares eight of the studies included in this review.

Furthermore, this review incorporates additional studies in which specific bacterial species were examined in greater depth to identify potential markers for future interventions, including *A. onderdonkii, P. goldsteinii, and F. nucleatum*. Classic probiotic bacteria typically share the ability to produce beneficial compounds for the gut, such as short-chain fatty acids and lactic acid. Interestingly, the effect of probiotic bacteria on leakage was inconsistent: two studies^15,17^ observed a decrease in lactobacilli associated with AL, whereas one study^12^ reported an increase of bifidobacteria in the AL group. Streptococci were associated with less leakage^13^, and *Streptococcus thermophilus* is often included in probiotic formulations. Although somewhat inconsistent across different probiotic taxa, probiotics, and lactobacilli in particular, were more frequently associated with the protected group and showed lower abundance in the AL group^15,17^. This is further supported by evidence from randomized controlled trials^22^, which have associated probiotic treatment with improved postoperative outcomes in colorectal cancer surgery.

The findings of this review highlight the potential for microbiome-based interventions in colorectal surgery. Preoperative modulation of gut microbiota using probiotics, short-chain fatty acid supplementation, antibiotics, or dietary interventions could reduce AL risk. Additionally, microbial biomarkers such as Lachnospiraceae abundance could guide risk stratification and personalized management strategies for high-risk patients.

The strengths of this review are that it includes a comprehensive search strategy, adherence to PRISMA guidelines^23^, and inclusion of both clinical and experimental studies. These approaches enhance the robustness of the findings and facilitate a balanced understanding of the role of the gut microbiome in AL. However, several limitations were identified. First, there was considerable heterogeneity among included studies regarding microbiome sequencing techniques and patient populations, which may have introduced variability. Second, functional profiling of microbial communities was limited, restricting insights into underlying mechanisms. Lastly, the relatively small sample sizes in some studies may have reduced the generalizability of findings.

To address current limitations, future studies should prioritize, apart from harmonizing the methodology, the following:

1. Functional profiling: investigating microbial pathways and mechanisms that influence anastomotic healing.
2. Interventional trials: evaluating the efficacy of microbiome-modulating therapies in reducing AL incidence through randomized controlled trials.
3. Large cohort/randomized studies: conducting multicentre studies with larger and more diverse populations to improve the generalizability of findings and lower the heterogeneity issues that have been plaguing previous small-scale trials and cohorts. Furthermore, using intraoperative instead of preoperative sampling may result in better evaluation of the microbiome at the time of anastomotic healing.

Future research should focus on functional profiling, microbiome-modulating interventions, and large, multicentre trials to validate these findings. Incorporating microbiome-based strategies into clinical practice could improve surgical outcomes and enhance recovery for patients undergoing colorectal cancer surgery.

## Supplementary Material

zrag005_Supplementary_Data

## Data Availability

The data set used in this study is available on request to the corresponding author.
