# Supplementary material for: Association of the gut microbiome to colorectal anastomotic leakage: systematic review
Source: BJS Open. 2026 Apr 8;10(2):zrag005. doi: 10.1093/bjsopen/zrag005 (PMC13065240; doi:10.1093/bjsopen/zrag005)
Supplement: zrag005_Supplementary_Data [file zrag005_supplementary_data.docx]

**Title: Association of the Gut Microbiome to colorectal Anastomotic Leakage: Systematic Review**

Peter H Cashin^1^, Sara Artursson^1^, Filip Sköldberg^1^, Åsa Melhus^2^

^1^Department of Surgical Sciences, Uppsala University, Uppsala, Sweden.

^2^Department of Medical Sciences, Section of Clinical Microbiology, Uppsala University, Uppsala, Sweden.

**Corresponding author.** Peter Cashin **ORCID ID** 0000-0003-3474-9450; **Twitter** @CashinPtr

**Supplementary Materials - Index**

| **Supplementary Appendixes** |  |
| --- | --- |
| Prospero Review Protocol | *pag. 2* |
| PRISMA 2020 checklist | *pag. 6* |
|  |  |

**Supplementary Appendixes**

**PROSPERO Registration Protocol**

**Review Title:**
The Association of the Gut Microbiome with Anastomotic Leakage Following Colorectal Surgery: A Systematic Review

**1. Review Question**

What is the association between gut microbiome composition, diversity, and function and the occurrence of anastomotic leakage (AL) after colorectal surgery?

**2. Background**

Anastomotic leakage (AL) is a severe and potentially life-threatening complication following colorectal surgery. Emerging evidence suggests that the gut microbiome may play a role in anastomotic healing. Specific microbial taxa and reduced microbial diversity have been implicated in patients who develop AL. However, a comprehensive synthesis of this evidence, including functional microbial profiles and causality from animal models, is lacking. This review aims to systematically assess studies that explore the link between the gut microbiome and AL.

**3. Objectives**

To systematically review and synthesize the evidence on:

- Differences in gut microbial diversity and taxa between patients with and without AL.
- Functional analyses of microbiome profiles associated with AL.
- Experimental evidence for causality in animal models.

**4. Eligibility Criteria**

**Inclusion Criteria:**

- Studies reporting microbiome sequencing (e.g., 16S rRNA, metagenomics) related to colorectal surgery.
- Studies with AL as a defined endpoint.
- Human studies or animal studies that include human-derived microbiome samples (e.g., fecal transplants).
- Peer-reviewed full-text articles.

**Exclusion Criteria:**

- Review articles, book chapters, editorials, protocols.
- Studies with exclusively animal data not involving human microbiome samples.
- Studies without microbiome sequencing.

**5. Information Sources**

- **Databases:** Google Scholar and PubMed
- **Other sources:** Manual cross-referencing of included study bibliographies.

**6. Search Strategy**

Google Scholar:
("colorectal surgery" OR "colon surgery" OR "rectal surgery" OR "anastomosis") AND ("gut microbiota" OR "gut flora" OR "intestinal microbiome" OR "microbial biomarkers") AND ("anastomotic leakage" OR "postoperative complications" OR "surgical outcomes") AND ("predictive biomarkers" OR "prognostic biomarkers")

PubMed:
“colorectal surgery anastomosis microbiome”

Initial search performed in November 2024.

**7. Study Selection**

- Title and abstract screening by two independent reviewers.
- Full-text review for eligibility.
- Discrepancies resolved by consensus.
- Screening results documented using PRISMA 2020 flow diagram.

**8. Data Extraction**

Two reviewers will extract:

- Study design and characteristics
- Number of patients, AL cases, controls
- Sequencing methods
- Microbial diversity (alpha and beta)
- Taxa differences
- Functional analyses (where applicable)
- Causal relationships from animal studies (if tested)

Extraction will be managed in Microsoft Excel.

**9. Risk of Bias (Quality) Assessment**

Each included study will be evaluated using the **Newcastle-Ottawa Scale** for observational studies, with scores recorded under Selection, Comparability, and Exposure domains.

**10. Strategy for Data Synthesis**

- Narrative synthesis of microbial diversity, taxa, and functional analysis results.
- Tabular summary of study characteristics and outcomes.
- Meta-analysis is not planned due to study heterogeneity (e.g., varying endpoints, sequencing methods).

**11. Analysis of Subgroups or Subsets**

Where applicable:

- Comparison by type of colorectal surgery.
- Differentiation between preoperative and postoperative samples.
- Analyses stratified by sequencing method (e.g., 16S rRNA vs. metagenomics).

**12. Dissemination Plans**

Findings will be submitted for peer-reviewed publication and may be presented at surgical or microbiology conferences.

**13. Keywords**

Gut microbiome, anastomotic leakage, colorectal surgery, microbial diversity, 16S rRNA, metagenomics, functional profiling

**14. Review Team and Roles**

- **Peter H. Cashin (Lead author)** – Review design, data screening, data extraction, manuscript writing.
- **Sara Artursson** – Co-reviewer for screening and data extraction.
- **Åsa Melhus** – Advisor on microbiome methods and data interpretation.
- **Filip Sköldberg** – Clinical advisor, surgical relevance.

**15. Funding Sources**

Partially funded by the Swedish Cancer Society (Cancerfonden), grant number: 24 0824.

**16. Conflicts of Interest**

None declared.

**17. Registration Timing**

The systematic review was initiated in November 2024, and this protocol was prepared for PROSPERO registration prior to final analysis.

| **Section and Topic** | **Item #** | **Checklist item** | **Location where item is reported** |
| --- | --- | --- | --- |
| **TITLE** | | |  |
| Title | 1 | Identify the report as a systematic review. | Title |
| **ABSTRACT** | | |  |
| Abstract | 2 | See the PRISMA 2020 for Abstracts checklist. | Checked |
| **INTRODUCTION** | | |  |
| Rationale | 3 | Describe the rationale for the review in the context of existing knowledge. | Intro  Paragr 3, pg3 |
| Objectives | 4 | Provide an explicit statement of the objective(s) or question(s) the review addresses. | Intro  Paragr3, pg3 |
| **METHODS** | | |  |
| Eligibility criteria | 5 | Specify the inclusion and exclusion criteria for the review and how studies were grouped for the syntheses. | Methods  Eligibilitiy, pg 4 |
| Information sources | 6 | Specify all databases, registers, websites, organisations, reference lists and other sources searched or consulted to identify studies. Specify the date when each source was last searched or consulted. | Methods  Search strat  Pg4 |
| Search strategy | 7 | Present the full search strategies for all databases, registers and websites, including any filters and limits used. | Methods  Search strat  Pg4 |
| Selection process | 8 | Specify the methods used to decide whether a study met the inclusion criteria of the review, including how many reviewers screened each record and each report retrieved, whether they worked independently, and if applicable, details of automation tools used in the process. | Methods  Data ext  Pg5 |
| Data collection process | 9 | Specify the methods used to collect data from reports, including how many reviewers collected data from each report, whether they worked independently, any processes for obtaining or confirming data from study investigators, and if applicable, details of automation tools used in the process. | Methods  Data ext  Pg5 |
| Data items | 10a | List and define all outcomes for which data were sought. Specify whether all results that were compatible with each outcome domain in each study were sought (e.g. for all measures, time points, analyses), and if not, the methods used to decide which results to collect. | Methods  Data ext  Pg5 |
|  | 10b | List and define all other variables for which data were sought (e.g. participant and intervention characteristics, funding sources). Describe any assumptions made about any missing or unclear information. | Methods Data ext  Pg5 |
| Study risk of bias assessment | 11 | Specify the methods used to assess risk of bias in the included studies, including details of the tool(s) used, how many reviewers assessed each study and whether they worked independently, and if applicable, details of automation tools used in the process. | Methods  Data ext  Pg5 |
| Effect measures | 12 | Specify for each outcome the effect measure(s) (e.g. risk ratio, mean difference) used in the synthesis or presentation of results. | Methods  Data ext  Pg 5 |
| Synthesis methods | 13a | Describe the processes used to decide which studies were eligible for each synthesis (e.g. tabulating the study intervention characteristics and comparing against the planned groups for each synthesis (item #5)). | Methods  Data ext  Pg 5 |
|  | 13b | Describe any methods required to prepare the data for presentation or synthesis, such as handling of missing summary statistics, or data conversions. | None used |
|  | 13c | Describe any methods used to tabulate or visually display results of individual studies and syntheses. | Only tables, no visual |
|  | 13d | Describe any methods used to synthesize results and provide a rationale for the choice(s). If meta-analysis was performed, describe the model(s), method(s) to identify the presence and extent of statistical heterogeneity, and software package(s) used. | No meta-analysis possible |
|  | 13e | Describe any methods used to explore possible causes of heterogeneity among study results (e.g. subgroup analysis, meta-regression). | Not applicable |
|  | 13f | Describe any sensitivity analyses conducted to assess robustness of the synthesized results. | Not applicable |
| Reporting bias assessment | 14 | Describe any methods used to assess risk of bias due to missing results in a synthesis (arising from reporting biases). | Methods  Data ext  Pg 5 |
| Certainty assessment | 15 | Describe any methods used to assess certainty (or confidence) in the body of evidence for an outcome. | Methods  Data ext  Pg 5 |
| **RESULTS** | | |  |
| Study selection | 16a | Describe the results of the search and selection process, from the number of records identified in the search to the number of studies included in the review, ideally using a flow diagram. | Flowchart |
|  | 16b | Cite studies that might appear to meet the inclusion criteria, but which were excluded, and explain why they were excluded. | None found |
| Study characteristics | 17 | Cite each included study and present its characteristics. | Table 1 and 2 |
| Risk of bias in studies | 18 | Present assessments of risk of bias for each included study. | Table 3 |
| Results of individual studies | 19 | For all outcomes, present, for each study: (a) summary statistics for each group (where appropriate) and (b) an effect estimate and its precision (e.g. confidence/credible interval), ideally using structured tables or plots. | Not applicable |
| Results of syntheses | 20a | For each synthesis, briefly summarise the characteristics and risk of bias among contributing studies. | Table 1 and 3 |
|  | 20b | Present results of all statistical syntheses conducted. If meta-analysis was done, present for each the summary estimate and its precision (e.g. confidence/credible interval) and measures of statistical heterogeneity. If comparing groups, describe the direction of the effect. | No meta-analysis |
|  | 20c | Present results of all investigations of possible causes of heterogeneity among study results. | Not applicable |
|  | 20d | Present results of all sensitivity analyses conducted to assess the robustness of the synthesized results. | Not applicable |
| Reporting biases | 21 | Present assessments of risk of bias due to missing results (arising from reporting biases) for each synthesis assessed. | Table 3 |
| Certainty of evidence | 22 | Present assessments of certainty (or confidence) in the body of evidence for each outcome assessed. | Table 3 |
| **DISCUSSION** | | |  |
| Discussion | 23a | Provide a general interpretation of the results in the context of other evidence. | Discussion – first paragraph |
|  | 23b | Discuss any limitations of the evidence included in the review. | Discussion limitations |
|  | 23c | Discuss any limitations of the review processes used. | Discussion limitations |
|  | 23d | Discuss implications of the results for practice, policy, and future research. | Discussion |
| **OTHER INFORMATION** | | |  |
| Registration and protocol | 24a | Provide registration information for the review, including register name and registration number, or state that the review was not registered. | Stated in methods |
|  | 24b | Indicate where the review protocol can be accessed, or state that a protocol was not prepared. | Stated in methods |
|  | 24c | Describe and explain any amendments to information provided at registration or in the protocol. | Not applicable |
| Support | 25 | Describe sources of financial or non-financial support for the review, and the role of the funders or sponsors in the review. | See funding |
| Competing interests | 26 | Declare any competing interests of review authors. | None to declare |
| Availability of data, code and other materials | 27 | Report which of the following are publicly available and where they can be found: template data collection forms; data extracted from included studies; data used for all analyses; analytic code; any other materials used in the review. | Available upon request |

*From:*  Page MJ, McKenzie JE, Bossuyt PM, Boutron I, Hoffmann TC, Mulrow CD, et al. The PRISMA 2020 statement: an updated guideline for reporting systematic reviews. BMJ 2021;372:n71. doi: 10.1136/bmj.n71
